# Bradykinin, insulin, and glycemia responses to exercise performed above and below lactate threshold in individuals with type 2 diabetes

**DOI:** 10.1590/1414-431X20176400

**Published:** 2017-09-12

**Authors:** R.Y. Asano, R.A.V. Browne, M.M. Sales, G. Arsa, J.F.V.N. Moraes, H.J. Coelho-Júnior, M.R. Moraes, I. Oliveira-Silva, S.E. Atlas, J.E. Lewis, H.G. Simões

**Affiliations:** 1 Curso de Educação Física, Universidade Ibirapuera, São Paulo, SP, Brasil; 2 Curso de Educação Física, Fundação Municipal de Educação Superior de Bragança Paulista, SP, Brasil; 3 Programa de Pós-graduação em Ciências da Saúde, Universidade Federal do Rio Grande do Norte, Natal, RN, Brasil; 4 Departamento de Educação Física, Universidade Estadual de Goiás, Quirinópolis, GO, Brasil; 5 Programa de Pós-graduação Stricto Sensu em Educação Física, Universidade Federal de Mato Grosso, Cuiabá, MT, Brasil; 6 Colegiado de Educação Física, Universidade Federal do Vale do São Francisco, Petrolina, PE, Brasil; 7 Programa de Pós-graduação em Educação Física, Universidade Estadual de Campinas, Campinas, SP, Brasil; 8 Programa de Pós-graduação Stricto Sensu em Educação Física, Universidade Católica de Brasília, Brasília, DF, Brasil; 9 Curso de Educação Física, UniEvangélica, Centro Universitário de Anápolis, Anápolis, GO, Brasil; 10 Department of Medicine, University of Miami Miller School of Medicine, Miami, FL, USA; 11 Department of Psychiatry and Behavioral Sciences, University of Miami Miller School of Medicine, Miami, FL, USA

**Keywords:** Metabolic disease, Aerobic exercise, Peptide, Insulin resistance

## Abstract

The aim of this study was to analyze the acute responses of bradykinin, insulin, and glycemia to exercise performed above and below lactate threshold (LT) in individuals with type 2 diabetes mellitus (T2D). Eleven participants with a diagnosis of T2D randomly underwent three experimental sessions 72 h apart: 1) 20 min of exercise performed at 120% of LT (120%LT), 2) 20 min of exercise performed at 80% of LT (80%LT), and 3) 20 min of control session. Blood glucose was analyzed before, during, and at 45 min post-exercise. Bradykinin and insulin were analyzed before and at 45 min post-exercise. Both exercise sessions elicited a parallel decrease in glucose level during exercise (P≤0.002), with a greater decrease being observed for 120%LT (P=0.005). Glucose decreased 22.7 mg/dL (95%CI=10.3 to 35, P=0.001) at the 45 min post-exercise recovery period for 80%LT and decreased 31.2 mg/dL (95%CI=18.1 to 44.4, P<0.001) for 120%LT (P=0.004). Insulin decreased at post-exercise for 80%LT (P=0.001) and control (P≤0.035). Bradykinin increased at 45 min post-exercise only for 80%LT (P=0.013), but was unrelated to the decrease in glucose (r=-0.16, P=0.642). In conclusion, exercise performed above and below LT reduced glycemia independently of insulin, but exercise above LT was more effective in individuals with T2D. However, these changes were unrelated to the increase in circulating bradykinin.

## Introduction

Blood glucose control is the top priority in the treatment of type 2 diabetes mellitus (T2D), as individuals with T2D and inadequate glycemic control are prone to chronic complications, such as hypertension, retinopathy, nephropathy, dyslipidemia, and other physiological impairments, thereby increasing morbidity and mortality in this population (1). Physical exercise has been extensively recommended for individuals with T2D due to its immediate and long-term effects in lowering blood glucose (2). Furthermore, individuals with T2D should perform aerobic exercise at least at moderate intensity, but higher intensity exercise may be even more effective (3,4).

Exercise can increase blood glucose uptake and its utilization by active skeletal muscle, even with low serum insulin levels. It can serve as an alternative means for glycemic control, since the insulin-dependent pathway is already impaired in T2D (5). However, an exercise-triggered, insulin-independent mechanism for glucose uptake has not yet been fully elucidated. Increasing calcium ions and the activation of pathways responsible for bradykinin release might be associated with blood glucose uptake in an insulin-independent pathway (6).

A classic study conducted by Taguchi et al. (7) demonstrated in rodents and humans the effectiveness of acute aerobic exercise in increasing the release and activity of bradykinin in parallel with blood glucose reduction in T2D. Despite the likelihood that exercise-induced bradykinin release would be associated with blood glucose reduction in T2D, to the best of our knowledge no studies have investigated the acute effects of exercise performed above and below lactate threshold on bradykinin, blood glucose, and insulin responses in this population.

We hypothesized that bradykinin release would be exercise intensity-dependent in individuals with T2D, which in turn would influence blood glucose control. In previous studies, we have demonstrated extensively that lactate threshold intensity, which distinguishes domains of moderate to high intensity exercise (8,9), may lead to distinct cardiovascular and metabolic responses during the post-exercise recovery period (10,11). Thus, the aim of the present study was to analyze the acute responses of glycemia, insulin and bradykinin to exercise performed above and below lactate threshold in individuals with T2D.

## Material and Methods

### Sample

Subjects participated in a controlled trial with a crossover design. After approval from the Research and Ethics Committee of the Universidade Católica de Brasilia (Protocol No. 167/2011) and signing of the informed consent form (Resolution 466/2012 of the Brazilian National Health Counsel and provisions of the Declaration of Helsinki), 11 individuals (6 women) from the city of Brasilia, clinically diagnosed with T2D participated in the study. Participants were non-smokers with a mean age of 62.1±9.0 years. The diagnosis of diabetes was confirmed with a medical evaluation using criteria from the American Diabetes Association (12). All individuals were under medical and nutritional treatment, using one oral hypoglycemic medication (e.g., sulfonylureas, metformin, glibenclamide plus metformin, glimepiride, pioglitazone hydrochloride) and following a diabetic diet to help control their blood glucose.

Exclusion criteria included a diagnosis of peripheral autonomic neuropathy, for which the following aspects were considered: 1) resting heart rate (HR) higher than 90 bpm, 2) incapable of reaching 85% of the age-predicted maximum HR during the maximal incremental exercise test (MIET), 3) a HR reduction less than 12 bpm during the first min after finishing the incremental test, and 4) abnormal HR variability (13). The participants could not have ulcers characteristic of diabetic foot or any other orthopedic impairment that could preclude performing exercise. Furthermore, the individuals could not have been on insulin or any other medicine that could interfere with the outcome variables to be evaluated.

### General procedures

All experimental sessions were performed in the Physical Evaluation and Training Laboratory at the Universidade Católica de Brasilia during the morning, 2 h after ingestion of a standard moderate glycemic index (GI=73.9) breakfast that provided a total of 315.9 kcal, 53 g (61.7%–212 kcal) of carbohydrate, 4.6 g (5.8%–18.3 kcal) of protein, and 9.5 g (27.1%–85.6 kcal) of fat. All medication was washed out for 24 h prior to the initial screening visit and the three subsequent sessions. The participants were also asked to avoid physical exercise and alcoholic or caffeinated drinks for 24 h prior to each visit to the laboratory. The participants also underwent a clinical evaluation including a resting electrocardiogram (ErgoPC Elite, Micromed, Brazil), blood pressure (BP) measurements (BP3AC1-1, Microlife Cooperation, Switzerland), anthropometry (body weight, height, and waist circumference), and a maximal incremental exercise test (MIET) on a cycle ergometer (Lode Excalibur Sport, The Netherlands). Body mass index (BMI) was calculated considering the ratio of body weight (2096 PP Standard, Brazil) in kilograms and height (Stadiometer 214, Seca, USA) in squared meters (kg/m^2^).

### Maximal incremental exercise test

Maximal heart rate (HR_max_), lactate threshold (LT), and peak oxygen uptake (VO_2peak_) were determined during the MIET. After conducting the clinical evaluation, participants performed the MIET on a cycle ergometer with an initial load of 15 W, followed by a 15 W increase at each 3-min stage at a speed of 60 revolutions per min until volitional exhaustion. During the MIET, a cardiologist monitored the electrocardiogram of the participants. HR, BP, rating of perceived exertion (RPE), ventilation, and blood lactate also were continuously monitored. The following criteria were used to determine whether participants achieved maximal effort: respiratory-exchange ratio (RER) ≥1.1, HR >90% age-predicted maximum, and RPE >17 (14).

Before exercise and during the last 20 s of each stage, 25 µL of capillary blood was drawn from the earlobe, and the samples were collected in Eppendorf microtubes containing 50 µL of 1% sodium fluoride (NaF) for analysis of lactate concentration, using the electro-enzymatic method (Yellow Springs 2.700 STAT, USA). Gas exchange during the MIET was obtained through the facemask of the Metalyzer 3B Gas Analyzer (Cortex Boiphysik, Germany) previously calibrated with a 3-L syringe (calibration flux) and a mixed pattern of gas containing 4.9% of CO_2_ and 17% of O_2_ (calibration gas). Ventilation, oxygen uptake (VO_2_), and carbon dioxide production (VCO_2_) were registered during the whole procedure, with the last 20 s of every 3-min stage being analyzed. In addition, BP was measured by the auscultatory method using a sphygmomanometer and a stethoscope (Tycos Hospital Instruments, Brazil) during the last 60 s of each 3-min stage. All equipment was calibrated according to manufacturers' instructions.

### Lactate threshold determination

To determine the LT, lactate concentration kinetics were examined during the MIET stages according to Simões et al. (9). Based on this previous report, LT was identified in individuals with T2D as an exercise intensity (Watts) above which the blood lactate concentration increases over-proportionally in relation to the workload increase. It is important to point out that LT intensity is similar to both blood glucose threshold and ventilatory thresholds (9).

### Experimental sessions (80 and 120% of LT intensity)

In these sessions, the volunteers performed 20 min of aerobic exercise on a cycle ergometer (Lode Excalibur Sport) with a relative intensity of 80% (80%LT: moderate intensity) and 120% (120%LT: high intensity) of the LT workload that was previously determined during the MIET. A fixed 20-min exercise was chosen to avoid effects of exercise duration on endocrine and perceptual physiological responses that are time-dependent, as previously described (15,16). The order of sessions [80%LT, 120%LT, and control (CON)] was randomized, and the minimum and maximum intervals between sessions were 72 and 120 h, respectively. To try to isolate the effect of exercise on metabolic activity, individuals were prevented from receiving any visual stimulus in the recovery period. Therefore, no activity, such as reading, was allowed during this period. Thus, individuals were instructed to remain seated until the 45th min.

### Control session

The control followed the same procedures applied in 80%LT and 120%LT. However, the participants remained seated in a resting position without performing exercise.

### Measurements performed in the experimental sessions

In all sessions, at 15 min of pre-exercise rest, a 25 µL blood sample was collected from the earlobe (glucose). Blood samples were taken every 5 min during the 20 min of either exercise (80%LT or 120%LT) or CON, as well as at 15 and 45 min of the post-session recovery period. Capillary blood samples were collected and analyzed similarly to blood lactate measures.

Furthermore, at 15-min of pre-exercise rest and at the 15th and 45th min of post-intervention recovery, a blood sample (8 mL) was collected from the antecubital or radial vein (bradykinin and insulin) and was deposited in two 4-mL tubes (Vacuette, BD, USA). Samples were centrifuged at room temperature for 15 min at 1509.3 *g* Plasma aliquots were separated in Eppendorf microtubes containing inhibitors of kinin degradation – orthophenanthroline, ethylenediamine tetraacetic acid (EDTA), dipyridyl, and sodium tetrathionate – and immediately stored at –20°C.

VO_2_, HR and lactate were monitored every 5 min (5th, 10th, 15th and 20th min) during the sessions and the average was calculated.

### Insulin

To measure serum insulin, we used human insulin enzyme-linked immunosorbent assay (ELISA) kit plate (Millipore). First, the wash buffer was diluted 10-fold by 450 mL of distilled water. After that, each well of the 96-well plate was filled with 300 μL of wash buffer and incubated for 5 min at room temperature (28°C). The assay buffer was then added to all control and sample wells except those that were used to generate the standard curve. Procedures were performed according to kit instructions.

### Bradykinin

Plasma bradykinin extraction was carried out using Sep-Pak C18 (Waters Corp., USA) columns previously activated with 90% acetonitrile (2 mL), water (5 mL), and 5% acetonitrile in 1% phosphoric acid (5 mL). Subsequent to the activation, the samples were applied in the column, washed with 5% acetonitrile in 1% phosphoric acid, and eluted at 35% acetonitrile in 1% phosphoric acid. The eluates were lyophilized, dissolved again in 500 µL of mobile phase A (5% acetonitrile in 0.1% orthophosphoric acid), and filtered through a 0.22 µm membrane for analysis by high performance liquid chromatography. The peptides were then separated on a reversed phase column (Aquapore 300 ODS; 250 × 4.6 mm), using isocratic gradient for 5 min followed by 20 min of linear gradient from 5% to 35% mobile phase B (95% acetonitrile in 0.1% H_3_PO_4_), under a flow of 1.5 mL/min for a period of 40 min. Bradykinin was identified by comparing the retention time with the standard kinin. All samples were tested in duplicate and the average values were considered.

### Statistical analysis

Data normality was tested using Shapiro-Wilk's test; skewness and kurtosis (z-score) were also tested. Descriptive variables are reported as mean±SD for parametric data and median and interquartile range (25th-75th percentile) for nonparametric data. Repeated measures ANOVA followed by Bonferroni's *post hoc* test was used to compare the values of VO_2_, blood lactate, %HR_max_, work rate, and area under the curve (AUC) of blood glucose, insulin and bradykinin levels between conditions. Friedman's ANOVA followed by pairwise comparison with Bonferroni's correction was used to verify the effect of time and condition on the blood glucose level during experimental conditions. The effect size of the *post hoc* test for nonparametric data was calculated by the formula r = z / √ *N*.

A two-way (condition *vs* time) repeated measures ANOVA was used to analyze blood glucose, insulin, and bradykinin levels following the experimental conditions. Partial eta squared (partial η^2^) was used to determine the effect size of the variance. In the case of a sphericity assumption violation, the degrees of freedom were adjusted and reported using the Greenhouse-Geisser epsilon correction. When necessary, Bonferroni's *post hoc* test was used to find significant differences. Spearman's correlation coefficient was used to examine the relationship between the absolute change in blood glucose and the absolute change in bradykinin in each experimental condition. The level of statistical significance was P<0.05. All data were analyzed using SPSS version 22.0 for Windows (IBM, Inc., USA).

The clinical significance was evaluated using an approach based on the magnitudes of change (17). Cohen's d was used to determine the effect size of absolute change between control and experimental sessions. The effect size magnitude was based in the Hopkins's scale: <0.2 is trivial, 0.2–0.5 is small, 0.6–1.1 is moderate, 1.2–1.9 is large and 2.0 or more is very large (18). For between-session comparisons, the chance that the true (unknown) values for each session were beneficial, unclear or harmful for change was calculated. Quantitative chances of beneficial or harmful effect were assessed qualitatively as follows: <1%, almost certainly not; 1–5%, very unlikely; 5–25%, unlikely; 25–75%, possibly; 75–95%, likely; 95–99%, very likely; and >99%, almost certainly. If the chances of having beneficial or harmful changes were both >10%, the true difference was considered unclear (17).

A *post hoc* statistical power analysis for the differences in the glucose responses between the three experimental conditions (i.e., pre- *vs* post-exercise) was conducted to determine the achieved power, based on the sample size (n=11), an alpha of 0.05, and the achieved effect size. For two-way repeated measures ANOVA, the achieved power for the interaction group by time was 92% and the main effect of time was 100%.

## Results

The main characteristics of the participants were 62.1±9.0 years old, body weight of 74.8±12.2 kg, height of 1.61±0.1 m, BMI of 28.8±4.6 kg/m^2^, waist circumference of 90.3±10.0 cm, fasting glucose of 154.7±56.8 mg/dL, VO_2_peak of 21.4±4.5 mL·kg^-1^·min^-1^, systolic blood pressure of 129.5±10.1 mmHg, and diastolic blood pressure of 73.1±10.3 mmHg. The mean fasting glucose value demonstrated a hyperglycemic condition, and the VO_2_peak revealed low physical fitness.

Table 1 shows the results of VO_2_, lactate, %HR_max_, exercise time and total work during the different experimental sessions (80%LT, 120%LT and CON), evidencing the differences of exercise intensities on studied parameters. Because a fixed 20-min exercise was chosen, not only the intensity but also total work was different between sessions. The mean results from all time-points of the 20-min experimental sessions revealed that VO_2_ was significantly higher in 120%LT compared to 80%LT and CON, and in 80%LT compared to CON (P<0.05). In addition, the % of maximal HR was different between exercise sessions (P<0.001). Furthermore, the final blood lactate measurement was higher only in 120%LT compared to CON, evidencing the higher exercise intensity (internal work) for the 120%LT.

**Table 1.** Mean values for VO_2_, blood lactate, % maximal HR and total work during the exercise (80%LT and 120%LT) and control conditions.

	CON	80%LT	120%LT
Time (min)	20	20	20
VO_2_ (mL·kg^−1^·min^−1^)	2.9±0.4	12.9±2.6^a^	17.6±3.0^a,b^
Lactate (mmol/L)	1.3±0.5	2.8±0.9	5.2±1.5^a^
HR_max_ (%)	46.5±6.9	77.5±7.1	92.6±11.1
Work (kJ)	93.1±20.8	352.1±91.8^a^	557.1±152.3^a,b^

CON: control session; 80%LT: exercise condition at 80% of lactate threshold; 120%LT: exercise condition at 120% of lactate threshold; VO_2_: oxygen consumption; HR_max_: maximal heart rate. ^a^P<0.05 compared to CON; ^b^P<0.05 compared to 80%LT (repeated measures ANOVA).

**Figure 1.**
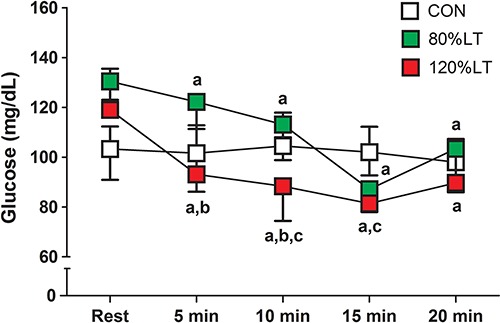
Blood glucose at each 5-min stage during the control, 80%LT, and 120%LT sessions. Data are reported as median, 25th and 75th percentiles). CON: control condition; 80%LT: exercise at 80% of lactate threshold; 120%LT: exercise at 120% of lactate threshold. ^a^P<0.0125 compared to rest at the same session; ^b^P<0.0167 compared to 80%LT at the same time; ^c^P<0.0167 compared to CON at the same time (Friedman's ANOVA).

Figure 1 shows the blood glucose levels during experimental conditions. Regarding 120%LT, the effect of time was significant (χ^2^(4)=31.6, P<0.001). *Post hoc* pairwise comparisons reveled that glucose decreased at the 5th (P=0.002, r=0.86), 10th (P=0.001, r=0.88), 15th (P=0.001, r=0.88), and 20th (P=0.001, r=0.89) min during exercise. Also, the effect of time was significant for 80%LT (χ^2^(4)=38.5, P<0.001), with *post hoc* pairwise comparisons revealing that glucose decreased at the 5th (P=0.001, r=0.89), 10th (P=0.001, r=0.88), 15th (P=0.001, r=0.88), and 20th (P=0.001, r=0.88) min during exercise. Regarding CON, the effect of time was significant (χ^2^(4)=9.7, P=0.04), but *post hoc* pairwise comparisons reveled no significant changes in glucose levels between rest and during exercise.

Regarding the effect of condition, the 5th min during exercise was significant (χ^2^(2)=6.7, P=0.04). *Post hoc* pairwise comparisons revealed that the decrease in glucose was higher in 120%LT compared to 80%LT (r=-0.80, P=0.005). For the 10th min, the effect was significant (χ^2^(2)=8.9, P=0.01) since *post hoc* pairwise comparisons revealed that the decrease in glucose was also higher in 120%LT compared to 80%LT (P=0.005, r=–0.80) and CON (P=0.007, r=–0.78). In the 15th min, the effect was also significant (χ^2^(2)=12.2, P=0.001), when the *post hoc* pairwise comparisons revealed that glucose decreased in 120%LT more than in CON (P=0.003, r=–0.83). Regarding the 20th min, the effect was marginally insignificant (χ^2^(2)=5.6, P=0.06).

**Figure 2.**
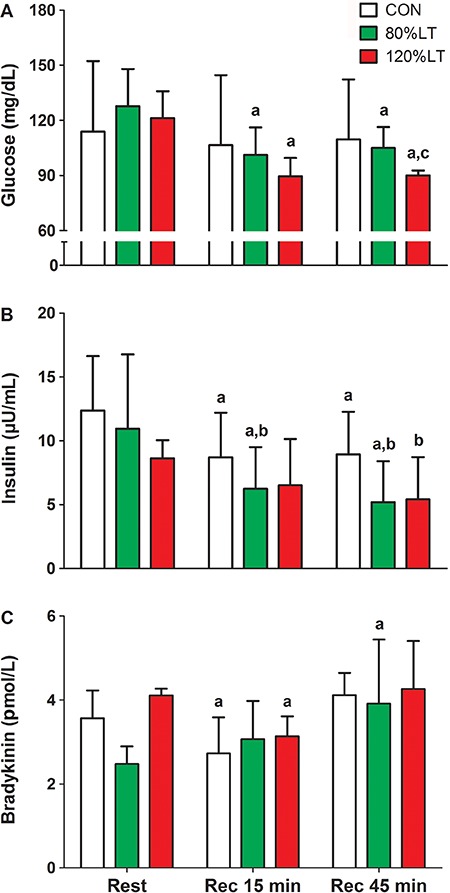
Blood glucose (*A*), insulin (*B*), and bradykinin (*C*) levels after the control (CON), 80%LT, and 120%LT sessions. Data are reported as means±SD. CON: control condition; 80%LT: exercise at 80% of lactate threshold; 120%LT: exercise at 120% of lactate threshold; Rec: recovery. ^a^P<0.05 compared to rest in the same session; ^b^P<0.05 compared to CON at the same time point; ^c^P<0.05 compared to 80%LT at the same time point of the session time (two-way ANOVA).

Figure 2 shows the blood glucose, insulin, and bradykinin levels at the 15th and 45th min of post-exercise recovery during the experimental sessions. For glucose, the main effect of time was significant (F(2, 20)=35.8, P<0.001, partial η^2^=0.78), and the condition × time interaction was also significant (F(2.6, 26.1)=6.3, P=0.003, partial η^2^=0.39). The effect of condition was non-significant (F(1.3, 12.9)=0.98, P=0.36, partial η^2^=0.09). Bonferroni's *post hoc* analysis revealed that glucose decreased at the 15th and 45th min of post-exercise recovery in 80%LT and 120%LT compared to rest in the same session (P<0.001). Additionally, the 120%LT session decreased more than the 80%LT after 45 min post-exercise (P=0.004).

Regarding insulin, the main effects of time (F(1.3, 12.8)=36.9, P<0.001, partial η^2^=0.79) and condition (F(2, 20)=7.8, P=0.003, partial η^2^=0.44) were significant. The condition × time interaction was also insignificant (F(2.2, 21.8)=1.6, P=0.223, partial η^2^=0.14). Bonferroni's *post hoc* analysis revealed that insulin decreased at the 15th and 45th min of post-exercise recovery in 80%LT (P=0.001) and CON (P≤0.04) compared to rest in the same session. Furthermore, the 80%LT session decreased more than CON at the 15th min of post-exercise recovery (P=0.03), and insulin decreased more at the 45th min in 80%LT (P=0.002) and 120%LT (P=0.004) compared to CON.

For bradykinin, the main effect of time (F(2, 20)=15.4, P<0.001, partial η^2^=0.61) and the condition × time interaction (F(4, 40)=4.5, P=0.005, partial η^2^=0.31) were significant. Bonferroni's *post hoc* analysis revealed that bradykinin decreased at the 15th min of post-exercise recovery in 120%LT (P<0.001) and CON (P=0.006) compared to rest in the same session. Also, bradykinin increased at the 45th min of post-exercise recovery only in 80%LT (P=0.013) compared to rest in the same session. In addition, the effect of condition was significant (F(2, 20)=6.7, P=0.006, partial η^2^=0.40). Bonferroni's *post hoc* analysis revealed that the bradykinin baseline level was different between CON and 80%LT (P=0.001) and between 80%LT and 120%LT (P<0.001).

Table 2 shows the clinical significance based in the magnitude of absolute change between control and experimental sessions. Regarding the main outcome (i.e., post-exercise blood glucose level), the 80%LT demonstrated a moderate effect size and was very likely of being clinically beneficial at both post-exercise time points. Furthermore, the 120%LT showed a moderate effect size and was very likely clinically beneficial at the first post-exercise time point and almost certainly clinically beneficial at the last post-exercise time point (i.e., 45 min post-exercise).

**Table 2.** Clinical significance following the exercise conditions.

	ES and 90% CL	Magnitude	Quantitative chance (%)			Qualitative chance
			Harmful	Trivial	Beneficial	
**Glucose**						
15-min post						
80%LT	−0.62 (−0.95 to −0.30)	Moderate	0%	2%	98%	Very likely
120%LT	−0.85 (−1.36 to −0.33)	Moderate	0%	2%	98%	Very likely
45-min post						
80%LT	−0.60 (−0.98 to −0.22)	Moderate	0%	4%	96%	Very likely
120%LT	−0.94 (−1.41 to −0.47)	Moderate	0%	1%	99%	Almost certainly
**Insulin**						
15-min post						
80%LT	−0.21 (−0.62 to 0.20)	Small	5%	44%	51%	Possibly
120%LT	0.43 (−0.19 to 1.04)	Small	74%	21%	5%	Possibly
45-min post						
80%LT	−0.46 (−0.90 to −0.01)	Small	1%	15%	84%	Likely
120%LT	0.06 (−0.61 to 0.74)	Trivial	36%	39%	25%	Unclear
**Bradykinin**						
15-min post						
80%LT	1.84 (0.94 to 2.75)	Large	0%	0%	100%	Almost certainly
120%LT	−0.25 (−1.23 to 0.74)	Small	53%	25%	21%	Unclear
45-min post						
80%LT	1.15 (0.16 to 2.13)	Moderate	2%	4%	94%	Likely
120%LT	−0.72 (−2.32 to 0.88)	Moderate	71%	12%	16%	Unclear

Effect size (ES) are Cohen's d (absolute change in control *vs* absolute change in exercise) and 90% confidence limits (90% CL). 80%LT: exercise at 80% of lactate threshold; 120%LT: exercise at 120% of lactate threshold.

**Figure 3.**
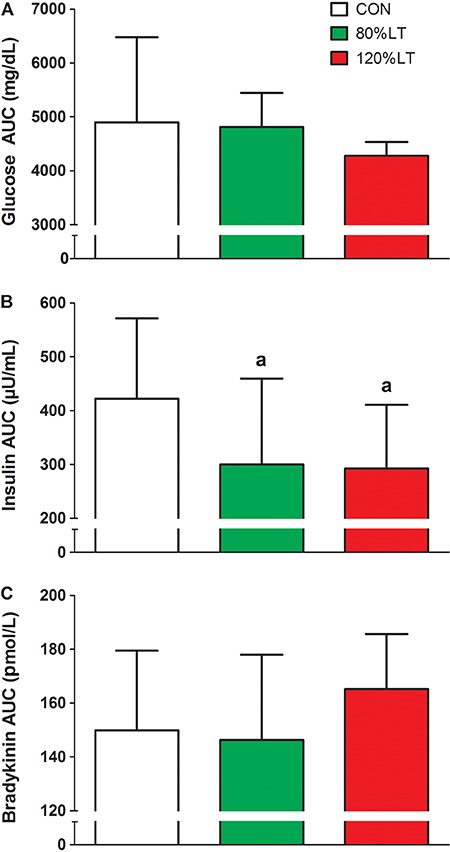
Area under the curve (AUC) of blood glucose (*A*), insulin (*B*), and bradykinin (*C*) after the CON, 80%LT, and 120%LT sessions. Data are reported as means±SD. CON: control condition; 80%LT: exercise at 80% of lactate threshold; 120%LT: exercise at 120% of lactate threshold. ^a^P<0.05 compared to CON (repeated measures ANOVA).

Figure 3 shows the total AUC between baseline and the 15th and 45th min of post-exercise recovery of glucose, insulin, and bradykinin levels following the experimental sessions. There was no effect of condition for glucose (F(1.3, 12.6)=1.52, P=0.248, partial η^2^=0.13) and bradykinin (F(2, 20)=2.07, P=0.152, partial η^2^=0.17). Regarding insulin, there was an effect of condition (F(2, 20)=8.94, P=0.002, partial η^2^=0.47). Bonferroni's *post hoc* analysis revealed that insulin decreased in 80%LT (P=0.024) and 120%LT (P=0.021) compared to the CON.

**Figure 4.**
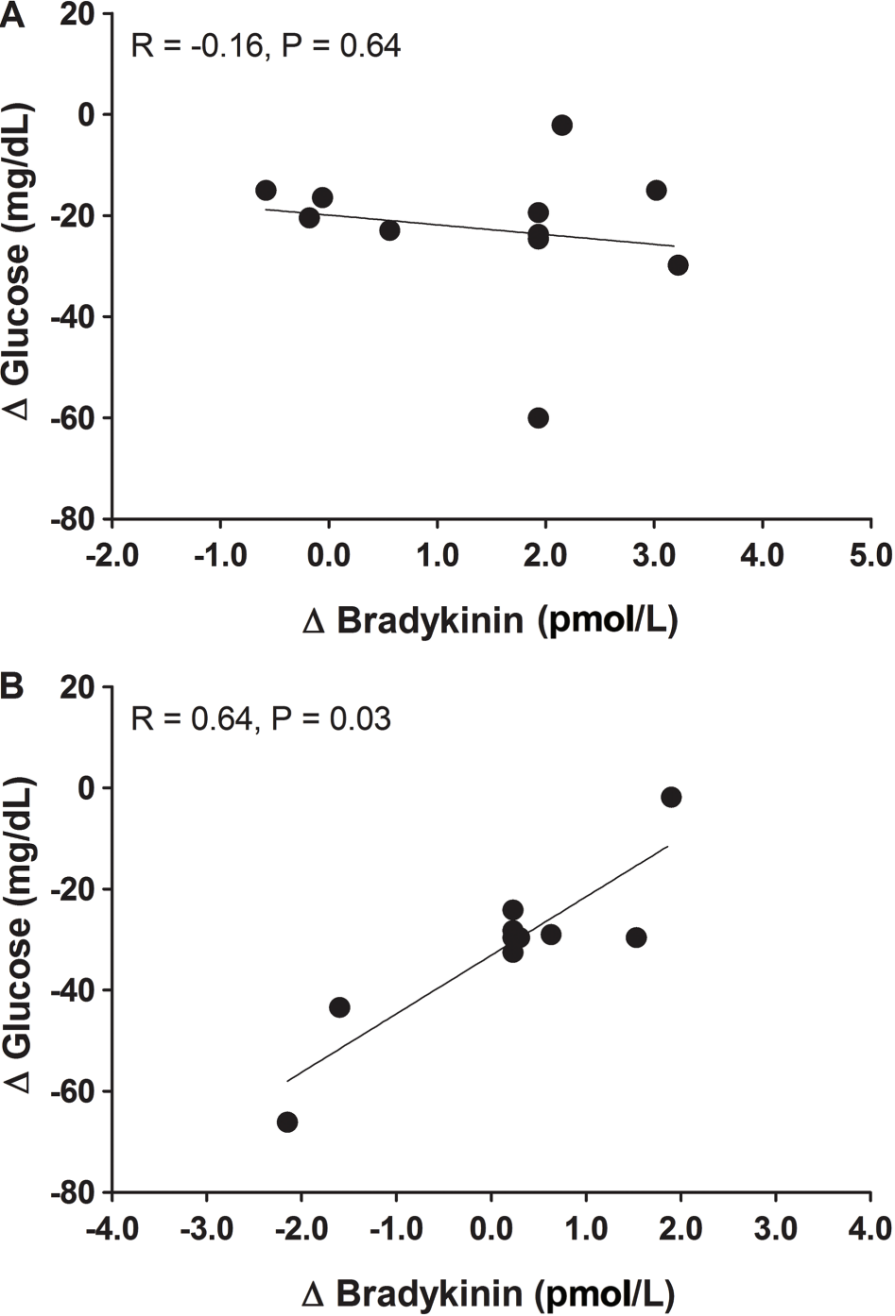
Spearman's correlation coefficient between absolute change [rec 45 min – rest] of blood glucose and bradykinin in the conditions of exercise at 80% of lactate threshold (*A*) and at 120% of lactate threshold (*B*).

Figure 4 shows the Spearman's correlation coefficient between delta values [rec 45 min – rest] of blood glucose and of bradykinin in the 80%LT and 120%LT conditions. The change in glucose was not related to the change in bradykinin in the 80%LT condition (r=-0.16, P=0.64). Regarding 120%LT, the change in glucose was significantly correlated with the change in bradykinin post-exercise (r=0.64, P=0.03).

## Discussion

The main finding of the present study was the effectiveness of aerobic exercise prescription based on LT intensity for individuals with T2D. The glycemia of participants decreased during and after exercise performed above and below the LT in T2D patients. However, the exercise session performed at 120%LT was more effective than 80%LT to decrease glucose during and after exercise, with no effect on total AUC for glycemia. The decrease in glycemia occurred with low insulin, suggesting a non-insulin dependent pathway of glucose uptake after exercise. In turn, bradykinin increased only 45 min after exercise performed at 80%LT, with no effects of exercise sessions on total AUC, and this was unrelated to the decrease in glucose. Thus, the null hypothesis of this study was supported, i.e., glucose uptake occurred without changes in plasma bradykinin in individuals with T2D.

The American College of Sports Medicine and the American Diabetes Association guidelines suggest that individuals with T2D perform aerobic exercise at moderate intensity (40–60% VO_2max_) (19). However, exercise at high intensity is encouraged, since it confers additional benefits (3,4). Meta-analytic data showed a higher potential for long-term high intensity exercise to increase VO_2max_ and decrease glycated hemoglobin compared to moderate exercise (3). Moreover, these alterations were not associated with the modulation of other exercise parameters, such as volume and frequency of exercise (3). However, no consensus exists about which exercise intensity can cause greater improvements in physiological systems (mainly on glucose level regulation) in individuals with T2D (3,19).

In the present study, exercise sessions performed in different exercise-intensity domains (i.e., above and below LT, eliciting distinct physiological responses as observed in Table 1) were selected based on their possible effects on glycemia. In previous studies from our research group, in which exercise intensities below and slightly above the LT (i.e., 10% below and 10% above LT) were analyzed (11,20), a blood glucose reduction was observed as exercise intensity increased. However, in the present investigation a higher intensity (20% above LT) was tested with wash-out medications.

The volunteers showed a greater decrease in blood glucose during and after exercise performed at 120%LT compared to 80%LT, and the clinical relevance was very likely to both intensities after 15 min post-exercise, but seemed to be more effective for 120%LT after 45 min of post-exercise recovery. However, in a previous study when ten individuals with T2D (56.9±11.2 years, 80.3±14.4 kg, 1.68±0.09 m, and VO_2max_ of 18.0±3.6 mL·kg^-1^·min^-1^) performed two sessions of exercise above and below LT, both sessions elicited comparable decreases in blood glucose (11). Similarly, Gross (21) did not observe any difference on glucose uptake among five young adults performing high-intensity interval exercise (80% VO_2max_) and low-intensity continuous exercise (60% VO_2max_). However, it is noteworthy that in both studies blood glucose was lower during the higher intense exercise (11,21). On the other hand, Boulé et al. (22) conducted a meta-analysis of 7 randomized clinical trials with a total sample of 266 individuals and found that intensity of exercise was strongly associated with the reduction of glycated hemoglobin (r=–0.91, P=0.002), whereas volume was not (r=–0.46, P=0.26).

Differences in experimental design (e.g., determination of LT and total diet kilocalories) is a possible explanation of the disagreement between the results of the present study and the results from Hiyane et al. (11) and Gross (21). Furthermore, the authors did not perform additional statistical analysis (e.g., effect size) that could have demonstrated the magnitude of the effect, which would better reflect the clinical significance of data (17).

As observed in Table 1, the exercise performed above the LT was of a greater total work (kilojoule) than that performed below LT, which might be pointed out as one limitation of the present study. While fixing the time of both sessions (20-min) to avoid influence of exercise time on physiological variables, the exercise sessions performed at different intensities (Watts) as related to LT (120 *vs* 80% LT) resulted in different total work. On the other hand, despite being of different work, this approach resulted in elevation in markers of exercise intensity (VO_2_, HR, blood lactate) that was significantly higher for the 120%, evidencing the effect of exercise intensity. The results shown in Figure 1 demonstrate that just 5-min of exercise performed at 120%LT was enough to reduce glycemia to values lower than that observed at same time points for 80%LT. Moreover, only 10-min of exercise at 120%LT was necessary for glycemia to be lower than control and 80%LT, while in the entire 20-min at 80%LT the glycemia was never statistically lower than control. Therefore, a one-time 10-min exercise at 120%LT (of which the amount of work was less than the 20th min exercise at 80%LT) was enough to reduce glycemia to values significantly lower than control. We assume that the main effect was due to exercise intensity, and not to the total work, because the glycemia at this point was reduced to values lower than control, and this was not observed even after the entire 20-min of exercise at 80%LT. Thus, it is reasonable to infer that physical exercise that requires more work per time promotes greater glucose uptake for patients with controlled diabetes, and without secondary complications such as the participants from this study.

Physical exercise leads to an increased AMP/ATP ratio, which activates AMP-activating protein kinase (AMPK) (23). AMPK is a protein that regulates certain pathways within cells, and its activation by physical exercise is not affected by T2D (24). In turn, when activated, AMPK elicits GLUT-4 translocation to sarcolemma, leading to glucose uptake (24,25). This way, it is possible that exercise performed at workload of 120%LT in the present study, leading to a higher recruitment of motor units and muscle mass activity, has elicited a more pronounced GLUT-4 translocation, resulting in a better blood glucose control of the participants. It has been shown in a previous study that activation of the involved signaling cascades is higher after exercise performed at 80% VO_2max_ than that of 40% VO_2max_, even when energy expenditure was equalized (400 kcal), resulting in an increase of AMKP (2.8-fold) and CaMKII phosphorylation (84%) immediately after the exercise of higher intensity (26).

Post-exercise glucose uptake can take place by insulin pathways due to effect of exercise increasing post-exercise insulin sensitivity (27). However, the effects of physical exercise on insulin seem to be dependent on exercise variables (e.g., intensity and duration) and subsequent diet (19). In the present study, serum insulin decreased after exercise, which implies that glucose uptake after exercise may have occurred due to both an increased post-exercise insulin sensitivity as well as by insulin-independent pathways.

Although not measured in the present investigation, studies have suggested that IL-6 plays a role in the glucose uptake process (28,29) and that its release is increased during exercise (30). Helge et al. (31) submitted 7 healthy men to three rectangular protocols of the knee extension (25, 65, and 85% of the maximum working capacity) and demonstrated that the higher the exercise intensity, the greater the release of IL-6 and, in turn, increased glucose uptake.

Bradykinin is a vasoactive peptide present in vascular smooth cells, endothelial cells, and in skeletal and cardiac muscles (32–34). Recently, kinin receptor (B1 and B2) knockout mice showed age-related hyperglycemia, increases of hepatic gluconeogenesis, intolerance of glucose, decreases in insulin sensibility, and functional impairment of pancreatic islets (35,36). This suggests a possible role of the kalikrein-kinin system in glucose uptake and homeostasis (35,36). In turn, Taguchi et al. (7) observed a blood glucose decrease in parallel with an increase in plasma bradykinin after exercise in a diabetic animal model. Moreover, researchers observed attenuation of both GLUT-4 translocation to the plasma membrane, IRS-1 phosphorylation, and PI3-K activity in skeletal muscle after infusion of a bradykinin antagonist-receptor (HOE-140) compared to a control group. This suggests a possible role of kalikrein-kinin system, mainly bradykinin, on glucose uptake by skeletal muscles (37).

In the present study, bradykinin increased only 45 min after exercise performed below LT, and differences were not observed between exercise sessions. In addition, the total AUC was not affected by exercise intensities, and was unrelated to the decrease of blood glucose. The effect of exercise intensity on bradykinin pathway was not evidenced in other studies in humans as well. Simões et al. (38) did not observe an increase in plasma bradykinin, kalikrein activity, or DesArg^9^-bradykinin after exercise performed at 90% of LT in T2D patients. These results corroborate trials developed in animal models (33,39). Schweitzer and Cartee (40) demonstrated that normal rats deficient in kininogen did not present differences in glucose transport after exercise. Other experiments developed by the same group did not show differences in blood glucose and insulin, nor in muscle glucose uptake, after exercise on B2 receptor knockout mice versus normal mice (39). Thus, it is reasonable to infer that bradykinin may not be essential for blood glucose uptake during and after exercise in T2D patients.

Another possible signaling pathway for glucose uptake, which could be a target of future investigations related to exercise intensity, is the AMPK activation from reductions in adenosine monophosphate/triphosphate and creatine/creatine phosphate ratios, or from the increase of the calcium/calmodulin complexes in skeletal muscle cells leading to signaling cascades of calcium/calmodulin protein kinases, or yet from activation of the mTOR/p70^S6K^ pathway, increasing GLUT-4 translocation to the surface of membrane, promoting glucose uptake (6). The most important limitation of this study is the lack of a control group without T2D, which could help explain the action of bradykinin on diabetic versus healthy individuals. The findings of this study may be important for exercise physiologists, health professionals involved in the treatment of T2D, scientists whose research focuses on blood glucose response in exercise training, and individuals with T2D who are interested in aerobic training.

In conclusion, aerobic exercise can decrease blood glucose during and after exercise performed above and below LT. However, exercise above LT demonstrated a greater blood glucose decrease than exercise performed below LT, and of a higher clinical relevance as evidenced by the effect size obtained post-exercise. The glycemia decrease occurred in parallel with an insulin decrease or stable insulin, suggesting possible involvement of insulin-independent pathways for glucose uptake during and after exercise. Bradykinin has been related to glucose homeostasis and glucose uptake, but in the present study, an increase of this peptide was observed only after 45 min of exercise performed below LT and was not related to the change in glucose. Thus, these results demonstrate that this peptide may not be the main factor that influences glucose uptake for T2D during and after aerobic exercise.
